# From decision to reflection: understanding the experiences and unmet care needs of patients treated with immunotherapy for melanoma in the adjuvant or metastatic setting

**DOI:** 10.1186/s12885-024-12410-7

**Published:** 2024-05-30

**Authors:** Nadia C.W. Kamminga, Astrid A.M. van der Veldt, Marlies Wakkee, Fauve R. van den Berge, Lianne A.A. van der Beek, Margot C.W. Joosen, Arjen Joosse, Karlijn de Joode, Tamar E.C. Nijsten, Marjolein Lugtenberg

**Affiliations:** 1 Department of Dermatology, Erasmus MC Cancer Institute, University Medical Center Rotterdam, Rotterdam, The Netherlands; 2 Department of Medical Oncology, Erasmus MC Cancer Institute, University Medical Center Rotterdam, Rotterdam, The Netherlands; 3 Department of Radiology and Nuclear Medicine, Erasmus MC Cancer Institute, University Medical Center Rotterdam, Rotterdam, The Netherlands; 4 Department Tranzo, Tilburg School of Social and Behavioral Sciences, Tilburg University, Tilburg, The Netherlands

**Keywords:** Melanoma, Skin cancer, Immune checkpoint inhibitors, Qualitative, Unmet care needs, Survivorship care

## Abstract

**Background:**

Despite increased use of immune checkpoint inhibitors (ICIs) in patients with advanced melanoma, little is known about patient experiences during this treatment. This study aimed to gain an in-depth understanding of experiences and unmet care needs of patients treated in the adjuvant or metastatic setting for advanced melanoma regarding their ICI treatment trajectory.

**Methods:**

Interviews and focus groups were conducted among 35 patients treated with ICIs in the adjuvant setting for completely resected stage III (*n* = 14), or in the metastatic setting for irresectable stage IV (*n* = 21) melanoma. A thorough thematic content analysis was conducted.

**Results:**

Three main themes were identified. When (1) *dealing with uncertainty in the decision-making process*, adjuvant patients explored the pros and cons, whereas metastatic patients considered immunotherapy their only viable option. Both groups expressed the need for additional guidance. In (2) *navigating the immunotherapy course*, both perceived the trajectory as intense, experienced a major impact on their and their (close) relatives’ lives, and felt the need to (re)gain control. When (3) *looking back on the immunotherapy experience*, metastatic patients generally felt relieved, while among adjuvant patients, feelings of doubt regarding their choice for ICIs were also reported.

**Conclusions:**

ICI treatment is perceived as intensive for both patient groups, facing both comparable and distinct challenges throughout the treatment trajectory, underscoring the need for stage-specific, individualised guidance. Options regarding flexible follow-ups, low-threshold contact and psychosocial support throughout the treatment trajectory should be explored.

**Supplementary Information:**

The online version contains supplementary material available at 10.1186/s12885-024-12410-7.

## Background

Immune checkpoint inhibitors (ICIs) have transformed the treatment landscape for patients with advanced melanoma [1]. ICIs, including anti-PD1- and anti-CTLA4-antibodies, stimulate the immune response by blocking the inhibitory synapse, and thereby activating T-cells [2]. Although initially reserved for irresectable stage III/IV, ICIs are increasingly used for treatment of earlier stages, i.e. the adjuvant setting of stage III [3] and II [4] melanoma. Particularly for stage III and IV, ICIs have shown substantial improvements in recurrence-free and overall survival, thereby potentially providing patients very durable responses and cure [1].

Although ICIs have been approved for treatment of advanced melanoma since over a decade, little is known about the experiences of patients. This treatment can be very intensive as it is associated with severe, potentially irreversible adverse events, frequent hospital visits and diagnostics. Previous quantitative [5–7] and few qualitative studies [8, 9] have examined the long-term impact of advanced melanoma and its treatment, showing that patients might experience serious side-effects and challenges regarding quality of life after discontinuing treatment. However, beyond the knowledge that they often experience uncertainties [10], little is known about the experiences of patients with stage III and IV melanoma *during* treatment: from decision-making to treatment discontinuation.

To support patients in coping with the impact of disease and treatment, the American Institute of Medicine (IOM) has been recommending survivorship care (SSC) for all cancer survivors after completing primary treatment [11]. Yet, previous research has shown that melanoma survivors’ SSC needs arise from diagnosis onwards [8, 12]. However, which specific needs patients have during treatment with ICIs remains unknown. This qualitative study aims to gain an in-depth understanding of the experiences and unmet care needs of patients treated in the adjuvant setting for stage III and in the metastatic setting for stage IV melanoma regarding their ICI treatment trajectory.

## Methods

### Study design and methodological considerations

A qualitative design was chosen to provide a rich description from the patients’ perspective [13]. Initially, we planned to conduct only focus groups to benefit from group dynamics [14]. However, in light of the COVID-19 pandemic, after the first focus group we decided to organise individual, telephonic interviews, providing a more personal and in-depth exploration of experiences [15].

The study’s reporting followed the Standards for Reporting Qualitative Research [16]. The study protocol was submitted to, and approved by, the Medical Ethics Committee Erasmus MC. After reviewing the protocol, the committee concluded that the Medical Research Involving Human Subjects Act (Dutch abbreviation: WMO) did not apply to this study (MEC-2019-0558 and MEC-2020-0197). Written informed consent was obtained from all participants involved in the study.

### Selection and recruitment of participants

Eligible participants for (group-)interviews were patients treated with ICIs in the adjuvant setting after complete resection of stage III melanoma (hereafter ‘patients in the adjuvant setting’), or in the metastatic setting for stage IV melanoma (hereafter ‘patients in the metastatic setting’) at the Erasmus MC in Rotterdam, the Netherlands, an academic hospital providing specialised skin cancer care. Using purposive sampling we aimed to capture maximum variation in experiences [17] based on sex, age, time since treatment discontinuation and perceived impact of ICIs, as assessed by their physician. Potential participants received an invitation letter and information leaflet detailing the study and participation process. Focus group and interview participants received compensation of a €40 (including travel expenses) or a €25 voucher respectively. Eventually, ten metastatic patients signed up to participate in the focus group, and after 12 additional interviews saturation was reached. For adjuvant patients, saturation was reached after a total of 14 interviews, and sampling of participants ended (see data analysis).

### Data collection

Before the (group-)interviews, patients’ demographics were collected through a short-self-administered questionnaire. Additional clinical characteristics, including disease stage, performance status, LDH level, presence of brain metastases, best tumour response [18], and reasons for treatment discontinuation, were extracted from patients’ electronic health records.

The focus group, moderated by two experienced focus group moderators (ML, female psychologist and MJ, female health scientist), was held at Erasmus MC in Rotterdam and lasted 120 min. The semi-structured interviews were conducted by three researchers (NK, female medical doctor and a male and female medical student) and took 50–90 min. None of them were directly involved in melanoma care. Discussions were structured using a topic guide which was developed based on existing literature concerning ICIs [19, 20] and experiences of the team (Appendix S1), addressing experiences with the treatment process and associated care needs. The topic guide was adjusted during analysis to emphasise decision-making. All discussions were audio-recorded.

### Data processing and analysis

The recordings were transcribed verbatim in anonymised form. All 27 transcripts underwent a thorough reflexive thematic content analysis [21], using NVivo version 12/R1®, incorporating elements from Grounded Theory i.e., different phases of coding, constant comparison and sampling until saturation [13, 22]. First, researchers summarised the transcripts, familiarising themselves with the data [21]. Subsequently, open coding of the first eight transcripts (four from each setting) was performed by one researcher (NK) and checked and complemented by a second (ML), resulting in two preliminary unstructured lists of open codes. During axial coding, relations between codes were identified, codes were grouped together, and (sub-)categories were created. The two resulting, more structured coding schemes were discussed within a multidisciplinary team (NK, ML and FB, female medical student) until consensus was reached. Two researchers (NK and FB) used these schemes to axially code the remaining 19 transcripts. In the final phase, selective coding, comparisons and contrasts between the two groups were also examined. The team (NK, ML, AV and FB) refined and named themes and core themes were identified, resulting in (partly overlapping) main- and sub-themes. Constant comparison was applied by comparing each interpretation and finding with new data [23]. Data saturation was reached for both groups when no more new concepts were identified [24]. A patient expert from each group reviewed and validated a draft version of the results.

## Results

Table 1 displays participants’ characteristics. A total of 14 patients in the adjuvant setting and 21 in the metastatic setting participated; 40.0% were female and 60.0% were male, with a median age of 56,4 years. Patients in the adjuvant setting started treatment 2–5 years ago, lasting a median of 8.1 months, with discontinuation reasons including completion of 12 months treatment, adverse events, patient’s request, the COVID-19 pandemic, or a combination. For the metastatic group, treatment started 2–9 years ago with a median duration of 11.5 months, reasons for discontinuation included stopping (per protocol) after 2 years, adverse events, complete or ongoing tumour response, or a combination. Treatment was still ongoing for two patients at the time of the interviews.

**Table 1:** Participants’ characteristics

Participant	Sex	Age (year)	Stage^a, b, c^	Performance status^a, d^	LDH^a^(U/L)	Brain metastases^a^	Treatment	Start of ICIs (year)	Best tumour response^e^	Recurrence	ICI duration (months)	Reasons discontinuation of ICIs
*Interview participants*												
Pt 1	F	39	IIIB	0	176	-	Nivolumab	2018	-	No	3	AE
Pt 2	M	61	IIIB	0	160	-	Nivolumab	2019	-	No	5	AE
Pt 3	M	65	IIIB	0	127	-	Nivolumab	2019	-	No	9	AE
Pt 4	M	58	IIIC	0	208	-	Nivolumab	2019	-	No	6	AE, RP
Pt 5	M	51	IIIB/C	0	168	-	Nivolumab/ pembrolizumab	2019	-	No	9	AE
Pt 6	M	56	IIIB	0	167	-	Nivolumab	2019	-	No	11	AE, CP
Pt 7	F	86	IIIC	0	188	-	Nivolumab	2019	-	No	11	CP
Pt 8	M	64	IIIC	0	145	-	Nivolumab	2019	-	No	12	TC
Pt 9	M	90	IIIC	0	101	-	Nivolumab	2019	-	No	3	AE
Pt 10	F	63	IIIB	0	176	-	Nivolumab	2019	-	No	12	TC
Pt 11	M	68	IIIB/C	0	196	-	Nivolumab	2019	-	No	12	TC
Pt 12	M	54	IIIB	0	188	-	Pembrolizumab	2020	-	No	5	AE, RP
Pt 13	M	80	IIIB	0	173	-	Nivolumab/ pembrolizumab	2020	-	No	8	AE
Pt 14	F	51	IIIA	0	218	-	Pembrolizumab	2021	-	No	7	AE
Pt 15	F	32	IV	0	125	No	Nivolumab	2018	PR	-	6	OR
Pt 16	M	78	IV	0	206	Unknown	Nivolumab	2017	PR	-	20	OR
Pt 17	F	45	IV	0	345	Unknown	Pembrolizumab	2016	CR	-	7	OR
Pt 18	M	72	IV	0	228	Unknown	Pembrolizumab	2016	PR	-	16	OR
Pt 19	F	38	IV	0	147	Unknown	Nivolumab	2018	PR	-	5	AE, OR
Pt 20	F	61	IV	0	151	No	Nivolumab	2017	CR	-	3	AE, OR
Pt 21	F	59	IV	1	440	Yes	Nivolumab	2019	PR	-	5	OR
Pt 22	M	48	IV	2	278	Yes	Ipilimumab/ nivolumab	2019	PR	-	-	-^f^
Pt 23	M	51	IV	1	1197	No	Ipilimumab/ nivolumab	2018	PR	-	24	TC, OR
Pt 24	F	40	IV	1	771	Yes	Ipilimumab/ nivolumab	2020	PR	-	-	-^f^
Pt 25	M	43	IV	0	184	Unknown	Nivolumab	2016	CR	-	12	OR
Pt 26	F	51	IV	0	284	No	Ipilimumab/ nivolumab	2021	CR	-	3	AE, CR
*Focus group participants*												
Pt 27	F	50	IV	0	147	Yes	Pembrolizumab	2014	CR	-	24	TC, OR
Pt 28	M	46	IV	0	158	No	Nivolumab	2017	CR	-	12	OR
Pt 29	F	51	IV	0	148	No	Nivolumab	2018	PR	-	6	AE, OR
Pt 30	M	37	IV	0	220	Yes	Ipilimumab/ nivolumab	2017	PR	-	24	TC, OR
Pt 31	M	56	IV	0	187	Unknown	Nivolumab	2017	CR	-	12	OR
Pt 32	M	53	IV	0	221	Unknown	Pembrolizumab	2016	CR	-	24	TC, OR
Pt 33	F	42	IV	0	200	Unknown	Pembrolizumab	2016	CR	-	24	TC, OR
Pt 34	M	83	IV	0	332	Unknown	Nivolumab	2016	PR	-	12	AE, OR
Pt 35	M	52	IV	1	264	No	Ipilimumab/ nivolumab	2018	PR	-	2	AE, OR

*LDH* Lactate dehydrogenase in U/L, *F* Female, *M *Male, *OR* Ongoing tumour response,* AE* Adverse events, *RP *Request of patient, *CP* COVID-19 pandemic, *TC* Treatment completed, *PR* Partial response, *SD* Stable disease, *CR *Complete response

^a^At start of ICIs

^b^According to the 8^th^ ed. Of the AJCC Melanoma Staging System [25]

^c^With all patients with stage III melanoma being treated in the adjuvant, and patients with stage IV melanoma being treated in the metastatic setting

^d^Assessed using World Health Organization (WHO) Performance Status criteria

^e^According to Response Evaluation In Solid Tumours (RECIST) 1.1 criteria [18]

^f^ICI ongoing, not yet discontinued

**Fig. 1:**
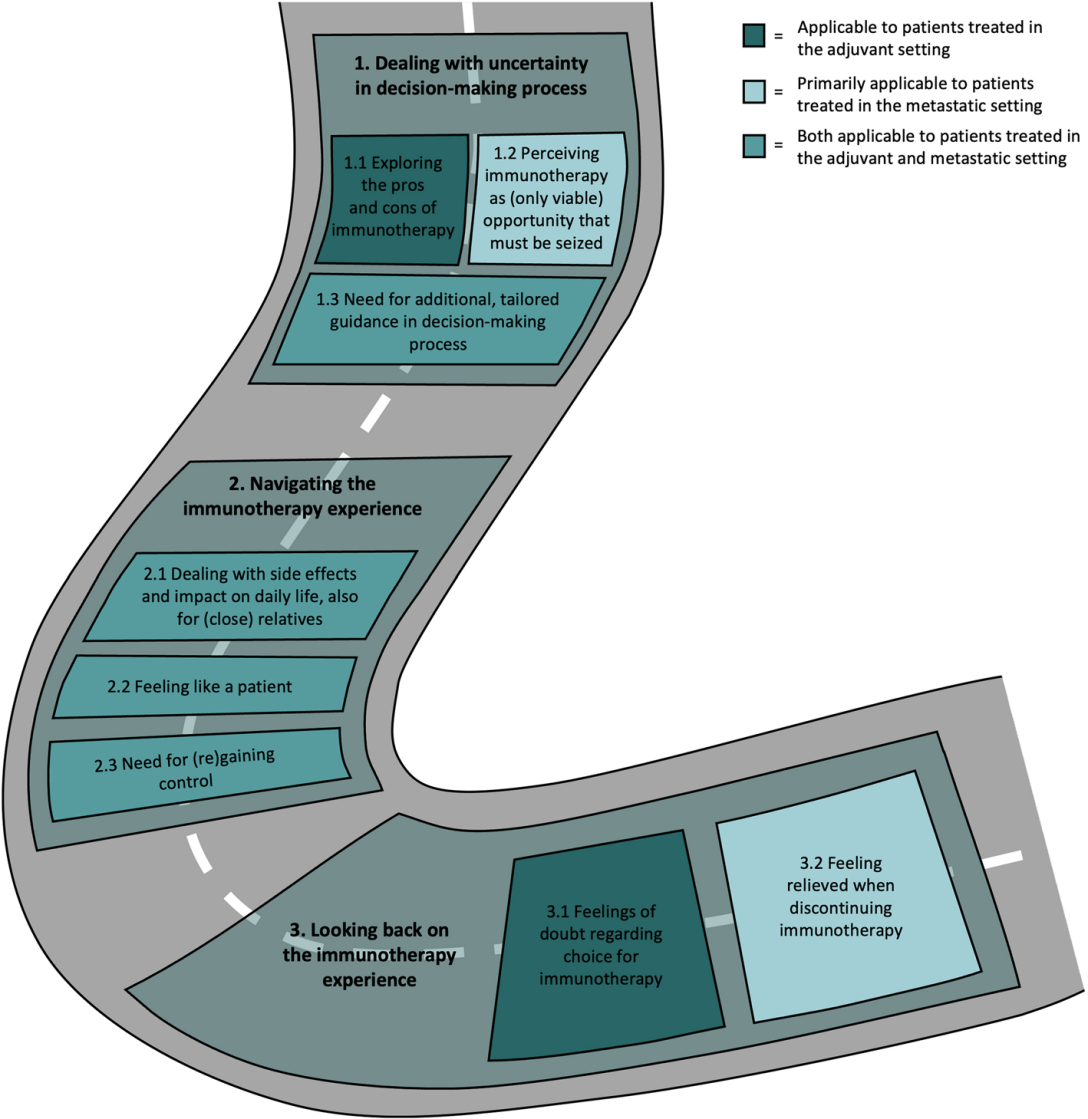
Overview of themes from decision to reflection

Analysis resulted in three main themes and eight subthemes (Fig. 1). Sub-themes apply to both groups, unless indicated otherwise.

### Dealing with uncertainty in decision making process

The first main theme identified was ‘dealing with uncertainty in decision making process’, which was subdivided into three subthemes.

#### Exploring the pros and cons of immunotherapy

This subtheme was only identified for patients in the adjuvant setting. When presented with the option for adjuvant immunotherapy or no adjuvant therapy, most patients experienced this as if they were presented with a clear choice for which they wanted to explore the pros and cons. Although having heard and read positive things about immunotherapy, they felt uncertain about its potential (influence on the) disease’s natural course, its effectiveness and, in particular, side effects. Patients often considered themselves healthy – or at least felt that way – after surgery and were afraid of the extensive list of potential problems and complaints.

*“The surgery, that was it for me really. So yeah […] having to make that choice, while I was actually healthy, I found that a bit tough”***– **Interview, adjuvant, female, 39 (Pt 1)

Patients often discussed their thoughts with their partners, friends and family, but also acquaintances having medical knowledge, weighing their opinions in their decision. Especially when having experienced something similar, patients felt truly understood by them and considered their opinions and advice valuable.

Although the decision-making time was perceived as short, accompanied with difficulties in weighing the provided information, the expected lower risk of recurrence and potential for better outcomes persuaded them to opt for treatment. Patients indicated that they would rather try and stop if needed, than experience regret afterwards. The doctor’s advice was mentioned as a decisive factor in making decision-making, and for some the ability to contribute to research also played a role.

#### Viewing immunotherapy as (only viable) opportunity that must be seized

This subtheme was predominantly identified for metastatic patients, though it also applied to those in the adjuvant setting. Most patients in the metastatic setting did not perceive receiving immunotherapy as an actual choice, viewing it as their only viable option to survive: patients were afraid of death and/or not finished with life and, according to them, there was no alternative and giving up was not an option.

*“What choice do you have? Either die, or go through treatment […] so no, starting that trajectory didn’t really feel like a choice”***– **Interview, metastatic, female, 51 (Pt 26)

These patients expressed gratitude to be eligible for immunotherapy – some even hoped they would be when receiving their diagnosis – and experienced this as an opportunity they had to seize to extend their lives, despite uncertainties about its effectiveness and timing. This sense of urgency, which was more prominent in the metastatic setting, led to a strong trust in their physicians’ recommendations. Consequently, they typically did not seek extensive input from their close relatives, often discussing their decisions more for confirmation than for advice. In addition, they expressed a lower demand for detailed information on potential side effects as they were committed to the treatment and did not wish to become excessively worried about potential outcomes.

#### Need for additional, tailored guidance in decision making process

Although patient recognised immunotherapy asa relatively new treatment, they stressed their need for more information about expected outcomes, the whole process around immunotherapy, and alternative options. Patients in the adjuvant setting expressed a need for greater awareness of potential side effects and more guidance making informed decisions. These patients received plenty of information on paper, but felt an additional conversation would have been useful. However, both groups emphasised the importance of information and guidance being tailored to individual needs, since not only the situation, but also needs may vary per patient.

*“A treatment is usually pretty personal, different for everyone. [...] I’m not interested in knowing everything. What applies to me, but not everything that’s on the internet”***– **Interview, metastatic, male, 48 (Pt 22)

While some found the support from close relatives sufficient, particularly valuing their presence at appointments as additional listeners, others suggested the ide a of a ‘buddy’ system. This would involve pairing new patients with someone that has gone through something similar from diagnosis onwards, to provide guidance and share insights throughout the treatment journey.

### Navigating the immunotherapy course

The second main theme identified was ‘navigating the immunotherapy course’, which consisted of three subthemes.

#### Dealing with side effects and impact on daily life, also for (close) relatives

Although also providing structure and stability, the intensity made patients feel as if they were being lived: they got on a rollercoaster they could not control. Patients emphasised many both physical and psychological side effects, including inflammations, itch, dry eyes, loss of taste and vitiligo, as well as fatigue and concentration problems. While some patients managed to continue their daily activities, using school and work as distractions, others had to work less or stop working altogether and had less energy for enjoyable activities, which sometimes felt like failure.

Patients also struggled dealing with reactions of those around them. Unlike their (close) relatives, patients did not necessarily share their enthusiasm about the positive effects of treatment: they only felt sicker because of it. Furthermore, the emotional toll and irritability sometimes led to conflicts at home. Despite these challenges, patients also expressed a heightened sense of being supported. However, the impact extended to their close relatives who often took on caregiving roles and sometimes had to cease working themselves.

Especially adjuvant patients stressed that they had seriously underestimated the treatments’ impact, finding the side effects more challenging than the cancer itself, which they found mentally challenging.

*“The weird thing is that… at that moment, you focus on your lungs (side effects). And, the fact that you have cancer, moves to the background.”***– **Interview, adjuvant, male, 61 (Pt 2)

Conversely, those in the metastatic setting noted that if these side effects had a major impact on their lives, they were willing to accept them because of the chance of survival and the possibility of growing older.

*“The side effects […] feeling nauseous, it’s just the way it is […] as for the nausea, it’s just a matter of what you can and can’t eat […] even if I can never have a steak again, I’ll just have chicken fillet. As long as I can just grow really old, I’m fine with it”***–** Interview, metastatic, female, 59 (Pt 21)

#### Feeling like a patient

The intensive treatment schedule with frequent hospital appointments, side effects and their impact on daily life (subtheme 2.1), elicited feelings of being a patient. They disliked being labelled or feel as patients: they either did not want to be sick or did not want to be treated as such, finding it more distressing than the disease itself. This feeling permeated beyond hospital walls, often brought up in social interactions, adding to their discomfort.

“*It’s a bit of a step back, you know? It’s not fun. And yeah, it’s okay in the hospital, but you can’t go anywhere without someone bringing it up. I really struggled with that. Actually, more than I did with being sick, to be honest”***– **Interview, metastatic, male, 43 (Pt 25)

Patients in the adjuvant setting expressed that starting treatment made them feel like they were actively becoming ill, particularly because they felt healthy and were symptom-free before starting treatment.

*“It felt like I was actively getting sick. Yeah, it’s really weird […] but, because of that infusion every two weeks, as a healthy person, visiting the hospital, all those scans, checks… while I was actually healthy”***– **Interview, adjuvant, female, 39 (Pt 1)

Within both stages however, not all patients were burdened by the patient label, or they at least did not let it affect their lives.

#### Need for (re)gaining control

During treatment, patients often felt overwhelmed and sought comfort in things they still had control over or could regain control of, such as flexible follow-ups, easy accessibility for contact, and the possibility for psychosocial support.

*“I started holding on to the things that I still had control over during that time. […] Like, things that I could still make decisions about, I just held on tight to those”***– **Interview, metastatic, female, 38 (Pt 19)

Furthermore, patients appreciated being able to influence the check-up frequency to better fit their lives, and to include loved ones in their hospital visits. Others, however, did not feel the need for having this influence, since they did not see the hospital visits as burdensome, but rather as a day out: to get a sandwich and to explore the city with their loved ones.

Patients also expressed a need to get quick answers to questions and concerns between these check-ups. Although they acknowledged that this is not possible, they expressed a desire to have daily contact with a doctor and emphasised their need for at least the option for more low-threshold telephone or email contact.

Additionally, patients emphasised a desire for actionable health tips and lifestyle advice to feel more involved in their recovery, and actively contribute to their health. This could contribute to their ‘fight against cancer’, a phrase that some found empowering, but others found annoying or rather saw it as a battle for their doctors. In addition, the need for psychosocial support e.g., from an occupational physician, psychologist or social worker during treatment was highlighted. They noted this support was underprovided, yet would have been beneficial from diagnosis onwards. Patients who were feeling and/or coping well, or considered the support from their loved ones sufficient (subtheme 2.1) had less need for this support.

### Looking back on the immunotherapy experience

The third main theme identified was ‘looking back on the immunotherapy experience’. This theme is subdivided into two subthemes, of which the first predominantly applies to patients in the metastatic setting, and the second theme solely applies to patients in the adjuvant setting.

#### Feeling relieved when discontinuing immunotherapy

Patients generally felt relieved upon discontinuing immunotherapy, despite the nervousness about stopping treatment in the metastatic setting where they considered it some sort of safety line (subtheme 1.2). Concerns about the impact of stopping, especially when having to because of serious side effects, coexisted with this relief. A good tumour response being visible on scans and sometimes even physically palpable added to that, providing a sense of security and confidence in a positive outcome.

*“Because I could feel the tumours under my skin, I could also feel them shrinking. And I have to say; that really helped. […] When you can feel them shrinking, that gives peace of mind”***- **Interview, metastatic, female, 40 (Pt 24)

Looking back, patients indicated the treatment process met their expectations, and remained positive about immunotherapy. This sentiment persisted even among patients who suffered side effects and they stressed they still stood behind their decision as they had weighed all pros and cons beforehand.

#### Feelings of doubt regarding choice for immunotherapy

Among patients in the adjuvant setting, feelings of doubt regarding their choice for immunotherapy were also reported, particularly when they asked to stop treatment earlier due to side effects and the desire not to be a patient anymore. They felt uncertain about the treatment effectiveness, as in this adjuvant setting no abnormalities were visible on scans or palpable, making the actual effect unclear. Despite understanding the potential benefits of preventing metastasis, some questioned whether the benefits truly outweighed the drawbacks. Although patients generally indicated they still stood behind their decision (subtheme 3.1), some expressed that, if presented with the same choice again, they would have chosen differently, prioritizing their quality of life over uncertain benefits.

*“No, with the white spots (vitiligo), I don’t think so. I wouldn’t have done it. I actually said to my wife last week: If I had to choose again whether to do it or not, I wouldn’t do it”***– **Interview, adjuvant, male, 54 (Pt 12)

## Discussion

This qualitative study shows that ICI treatment for melanoma in both the adjuvant and the metastatic setting is perceived as intensive and presents comparable and distinct challenges throughout the trajectory, translating in several unmet SSC needs. This asks for both stage-specific and individually tailored information and guidance.

The decision-making experiences of patients in the adjuvant versus the metastatic setting when considering ICI therapy show significant contrasts. Patients in the adjuvant setting often find this process challenging and seek extensive information, requiring more time for contemplation, and often discussing their choice with others. Conversely, patients in the metastatic setting feel compelled to make swift decisions due to the critical nature of their condition [26], regarding ICI treatment as a crucial lifeline and not a choice, but a necessity for survival. These differences between, but also within the groups underscore the vital need for stage-tailored and individualised information provision and guidance. For patients in the adjuvant setting introducing an additional consultation e.g., to answer questions and discuss concerns and uncertainties and/or the development of a decision tool might be valuable.

However, our results also showed similarities, with both groups experiencing a significant impact on their lives during treatment, from side-effects to the intensity of frequent clinic visits. Previous research indicates that both patients and their partners [27] often enter a survival mode during cancer treatment, with the real emotional toll manifesting afterwards [28]. However, our findings suggest that the impact can be substantial even during treatment, emphasising the need to refine survivorship care. Especially patients in the adjuvant setting underestimated this impact, and felt like they were actively becoming ill by ICI treatment. Patients in the metastatic setting could, even if having a great impact on their lives, accept the negative effects of treatment more easily. To support both groups in regaining control during treatment, a flexible check-up frequency, low-threshold access to the treatment team, and readily available psychological support throughout the treatment trajectory should be considered. Given the existing high workloads and staff shortages in healthcare, optimal ways for achieving this should be explored, potentially with the assistance of nurse practitioners or specialised oncology nurses.

Another important finding pertains to reflections on ICI treatment, where feelings of doubt were mentioned among patients treated for stage III melanoma. Decisional regret was previously described in research focusing on other types of cancer like prostate and breast cancer [29, 30] and a recent study of Atkinson et al. [31] highlighted its presence among patients with stage III melanoma who opted for adjuvant ICI treatment. Our study is the first to provide a qualitative description of these feelings. Some patients felt that the often unexpected and sometimes permanent side-effects did not weigh up to the often-unmeasurable treatment effectiveness. This underscores the importance of enhanced, tailored information and guidance during decision-making, especially since concerns regarding expected adverse events and unclear benefits of treatment are important factors during this process [32, 33]. Atkinson et al.‘s findings further demonstrate the complexity by revealing that patients informed via stage-tailored videos, predominantly (59%) chose observation, yet they experienced more decisional regret compared to those opting for adjuvant therapy [31]. This further underscores the importance of shared decision making (SDM) [34], that involves clear communication of choices, including benefits and harms, but also presentation of alternatives, and consideration of the patients’ values, preferences and circumstances [35, 36]. After all, if patients genuinely feel they have made a choice, they are more likely to confidently stand behind their decision [37].

One potential way to better equip patients and circumvent feelings of doubt or decisional regret is paring them with a “buddy” from diagnosis onwards: someone who has treaded a similar path, thereby offering invaluable peer-to-peer support [38] which could foster patient empowerment and thereby informed decision [38, 39]. Moreover, this could contribute to the aforementioned need for low-threshold contact and psychological support throughout the treatment trajectory [40]. In addition, this detailed description of experiences could serve as a rich resource itself.

Our study boasts several strengths. Foremost, this study is, to our knowledge, the first to provide an in-depth description of experiences and needs of patients undergoing ICI treatment for melanoma in both the adjuvant and metastatic setting. By employing a combination of individual interviews and a focus group with a variable sample of patients, we effectively combined the strengths of both methods while minimising their limitations [14, 15]. The strategy of capturing maximum variation in experiences has yielded rich data which, coupled with a thorough thematic content analysis [21], contributes to the robustness of our results.

However, there are also some limitations to consider. The COVID-19 pandemic might have influenced patients’ daily experiences, potentially accentuating negative experiences reported, particularly among those having doubts about ICIs. That said, our sample included only stage IV patients having a positive (partial or complete) response to treatment, whereas patients with negative outcomes may have unique needs and potentially higher levels of doubt or regret. Moreover, to understand the full spectrum of the decision-making process and to provide optimal guidance, experiences of patients who opted against treatment should be investigated. These vital perspectives remain unexplored in our study, stressing the need for future research focusing on these specific patient groups. Finally, our project is region-specific and limited to participants from one academic hospital. However, qualitative research is always context-specific [13]. Nevertheless, the identified themes may have broader transferability, especially in settings with similar melanoma care organisation.

In conclusion, this study highlights the intense trajectory for patients with melanoma receiving ICIs, whether in the adjuvant or metastatic setting, revealing differences but also similarities. In the adjuvant setting, patients adopted a broad perspective to decision-making, gathering more information, needing more time to do so and discussing their choices with others. Conversely, those in the metastatic setting perceived ICI treatment as critical lifeline, providing them with a necessary sense of security, indicating the need for stage-specific, individually tailored information and guidance in decision-making. To effectively support both patient groups in regaining control a tailored approach is needed, for which options regarding flexible follow-ups, easy accessibility for contact, and availability of psychosocial support even during treatment should be explored. This person-centred approach may significantly enhance patients’ experiences, wellbeing and empowerment throughout and beyond their ICI trajectory.

### Supplementary Information

Supplementary Material 1.

## Data Availability

The data that support the findings of this study are available on request from the corresponding author. The data are not publicly available due to privacy or ethical restrictions.

## Abbreviations

AE: Adverse events
CP: COVID-19 pandemic
CR: Complete response
ICI: Immune checkpoint inhibitor
LDH: Lactate dehydrogenase in U/L
OR: Ongoing tumour response
PR: Request of patient or partial response
SSC: Survivorship care
SDM: Shared decision making
SD: Stable disease
TC: Treatment completed
