# Supplementary material for: From decision to reflection: understanding the experiences and unmet care needs of patients treated with immunotherapy for melanoma in the adjuvant or metastatic setting
Source: BMC Cancer. 2024 May 30;24:662. doi: 10.1186/s12885-024-12410-7 (PMC11141069; doi:10.1186/s12885-024-12410-7)
Supplement: Supplementary file 1 — Supplementary Material 1. [file 12885_2024_12410_MOESM1_ESM.docx]

**Focus group (stage IV melanoma – metastatic setting)**

Introduction

- Background and aim of focus group study
- Structure of the focus group
- Informed consent, audio-taping and demographic questionnaires

Experiences with treatment process: period of diagnosis and period of treatment

- Experiences with diagnosis
- Experiences with immunotherapy

Experiences after discontinuation of immunotherapy

- Experiences after ending treatment
- ‘Zwarte gat’ animation/short movie and reflection

Experiences, preferences and needs regarding follow-up/supportive care and support throughout the trajectory

- Check-ups
- Counselling/information
- Support
- Follow-up/supportive care in general
- Survivorship care plan (SCP)

**Interviews (stage III melanoma – adjuvant setting)**

Introduction

- Background and aim of the study
- Structure of the interview
- Informed consent, audio-taping and demographic questionnaires

Diagnostic trajectory

- Experiences with receiving the diagnosis
- Experienced symptoms and overall impact

Experiences with immunotherapy

- Experiences with receiving metastastic diagnosis
- Treatment decision making
- Expectations and experiences with immunotherapy
- Side effects and general impact
- The end of and looking back on the period of immunotherapy (incl. reflecting on decision regarding immunotherapy)
- Uncertainty regarding long-term effects

Experiences after discontinuation of immunotherapy

- Problems they (continue to) encounter
- Positive experiences
- Total impact of melanoma
- Being a patiënt
- ‘Zwarte gat’ animation / short movie reflection

Experiences and needs regarding follow-up/supportive care and support throughout the trajectory

- Check-ups
- Counselling/information
- Support
- Follow-up/supportive care in general
- Survivorship care plan (SCP)

**Interviews (stage IV melanoma – metastatic setting)**

Introduction

- Background and aim of the study
- Structure of the interview
- Informed consent, audio-taping and demographic questionnaires

Diagnostic trajectory

- Experiences with receiving the diagnosis
- Experienced symptoms and overall impact

Experiences with immunotherapy

- Experiences with receiving metastastic diagnosis
- Decision making
- Expectations and experiences with immunotherapy
- Side effects and general impact
- The end of and looking back on the period of immunotherapy
- Uncertainty regarding long-term effects

Experiences after discontinuation of immunotherapy

- Problems they (continue to) encounter
- Positive experiences
- Total impact of melanoma
- Being a patiënt
- ‘Zwarte gat’ animation / short movie reflection

Experiences and needs regarding follow-up/supportive care and support throughout the trajectory

- Check-ups
- Counselling/information
- Support
- Follow-up/supportive care in general
- Survivorship care plan (SCP)
